# Anxiety symptoms among Chinese nurses and the associated factors: a cross sectional study

**DOI:** 10.1186/1471-244X-12-141

**Published:** 2012-09-14

**Authors:** Yu-Qin Gao, Bo-Chen Pan, Wei Sun, Hui Wu, Jia-Na Wang, Lie Wang

**Affiliations:** 1 Department of Nursing, Stomatology Hospital, China Medical University, No.117, Nanjingbei Street, Heping District, Shenyang, 110002, China; 2 Shengjing Hospital, China Medical University, No.36, Sanhao Street, Heping District, Shenyang, 110004, China; 3 School of Public Health, China Medical University, No.92, Bei’er Road, Heping District, Shenyang, 110001, China

## Abstract

**Background:**

Nurses are an indispensable component of the work force in the health care system. However, many of them are known to work in a stressful environment which may affect their mental well-being; the situation could be worse in rapidly transforming societies such as China. The purpose of this study was to investigate anxiety symptoms and the associated factors in Chinese nurses working in public city hospitals.

**Methods:**

A cross-sectional survey was performed for Chinese nurses in public city hospitals of Liaoning Province, northeast China. Seven hospitals in different areas of the province were randomly selected for the study. The Zung Self-Rating Anxiety Scale was used to measure anxiety symptoms. Effort-reward imbalance questionnaire and Job Content Questionnaire were used to assess the work stressors. Univariate analysis and stepwise multivariate logistic regression analysis were used to identify the factors associated with anxiety symptoms.

**Results:**

All registered nurses in the seven city hospitals, totaling 1807 registered nurses were surveyed. Of the returned questionnaires, 1437 were valid (79.5%) for analysis. Utilizing the total raw score ≥ 40 as the cut-off point, the prevalence of anxiety symptoms in these nurses was 43.4%. Demographic factors (education, chronic disease and life event), lifestyle factors (regular meals and physical exercise), work conditions (hospital grade, job rank, monthly salary, nurse-patient relationships, job satisfaction and intention of leaving), job content (social support and decision latitude), effort-reward imbalance and overcommitment were all significantly related to the anxiety symptoms. Multivariate logistic regression analysis showed main factors associated with anxiety symptoms were lower job rank (OR 2.501), overcommitment (OR 2.018), chronic diseases (OR 1.541), worse nurse-patient relationship (OR 1.434), higher social support (OR 0.573), lower hospital grade (OR 0.629), taking regular meals (OR 0.719) and higher level of job satisfaction (OR 0.722).

**Conclusions:**

A large proportion of Chinese nurses working in public city hospitals had anxiety symptoms, which warrants immediate investigation and intervention from the hospital administrators. Meanwhile, results of the study suggest that proper counseling, promotion of healthy lifestyle behavior and improvements to the social environment in the work place may be helpful toward reducing or preventing the anxiety symptoms.

## Background

Anxiety is a psychological and physiological state characterized by cognitive, somatic, emotional, and behavioral components which combine to create an unpleasant feeling that is typically associated with uneasiness, apprehension, fear, or worry [1]. Anxiety can be a normal reaction to stress or threat, and it may help one to deal with stressful or threatening situations. However, when it becomes excessive and persistent, it becomes a disabling medical condition known as anxiety disorder, which, if let untreated, can get worse; frequently accompanied by physiological symptoms such as headache, sweating, muscle spasms, palpitations, fatigue or even exhaustion [1].

Anxiety disorders are the most common class of psychiatric disorders in the U.S. [2] and many other countries [3]. In the U.S. alone, anxiety disorders affect about 40 million adults aged 18 years and older (about 18% of that population) annually and affect approximately 28.8% of the U.S. population in their life time [2,4]. According to an ESEMeD study including six European countries, the 12-month prevalence of inappropriate anxiety was 6.4% [5]. In a more recent systematic review of studies conducted in 16 European countries, however, this value was estimated to be 12% [6]. In China, anxiety is also one of the most common mental disorders. A recent survey of mental disorders in Chinese population aged 18 and older showed that the current (1-month) prevalence of anxiety disorders was about 5.6% [7]. It is estimated that one-eighth of the total population worldwide suffers from inappropriate anxiety [8].

Anxiety disorders adversely affect a person’s quality of life, and can be debilitating in severe cases. They can be symptoms of an underlying health issue such as chronic obstructive pulmonary disease (COPD) and/or heart failure [9]. Anxiety disorders are also highly comorbid with each other and other psychiatric disorders such as depression and substance abuse [4]. Many epidemiologic studies have suggested that psychiatric and nonpsychiatric patients with chronic anxiety may be at risk for developing coronary heart disease [10]. Anxiety disorders impose both an individual and a social burden that amounts to a total cost of $42.3 billion in the U.S. in 1990 [11]. On the other hand, population-based studies have shown that anxiety disorders frequently go untreated [12,13], and people with anxiety symptoms or disorders rarely seek help from mental or non-mental medical professionals [7].

Prevalence of anxiety disorders varies among different populations. For example, women have been known to have higher prevalence than men; Older age (e.g. 40 years or older) has been associated with higher prevalence than the younger; People living in urban areas tend to have higher prevalence than people in the rural areas [7]. In an UK obese population, as many as 56% of patients met the minimum criteria for an anxiety disorder [14]*.*

Nurses are an indispensable component of the work force in the health care system, and their work performance will undoubtedly affect the overall quality of patient care in the hospital. In this sense, the mental health of nurses deserves attention from nursing managers and hospital administrators. Studies have shown that, in general, the prevalence of anxiety in nurses is higher than that of the whole population, although it may vary greatly from country to country or between different nursing specialties. The lowest reported prevalence (7%) was seen in Japanese nurses who were working in the acute care hospitals (including departments of internal medicine, surgery, intensive care, operating room and outpatient clinic) [15]. The highest reported prevalence of anxiety (43.2%) was found in Iranian hospital nurses who had to do shift work [16]. In Singapore, 21% of nurses in a general hospital were found to suffering from anxiety disorders, but, interestingly, only a very small proportion actually sought help for their emotional problems (<4%) [17]. In the U.S., about 20% of ICU and general care nurses from different hospitals had symptoms consistent with possible anxiety [18]. Results of these studies indicated that hospital nursing is a profession that predisposes the workers to mental impairment such as anxiety.

Chinese nurses in the hospitals are known to work in stressful environment. This of course involves the characteristics of the profession such as the concerns with the occupational hazards (e.g. infections or injuries); worries over potential medical accidents and the associated consequences; physical and psychological exhaustion due to the intensive care of patients in emergency and seeing the death and suffering of patients. Social aspects of their work may also contribute to work stress. Generally, in Chinese society, the social status of nurses is relatively low and their work is often not highly valued. In some hospitals, they are not even well respected by their hospital administrators and doctors. In addition, the mode of nursing care in Chinese hospitals has changed rapidly in recent years. There is also dramatic reform in the medical care services in China which deviates from the previous market-oriented service system and emphasizes more the public welfare of the state owned hospitals. All the situations and changes make nursing practice even more demanding and challenging to nurses. As studies have pointed out the link between adverse psychosocial factors in the work environment and the psychological and physical health in nurses of other countries [19,20], we hypothesize that Chinese nurses in public hospitals may find it difficult to adapt to the stress in their work and, as a result, may develop mental health problems such as anxiety. To test the hypothesis, we conducted a survey on the anxiety symptoms, one of the most common of mental problems in Chinese nurses.

In order to explore the associated factors for the anxiety symptoms of Chinese nurses, two theoretical models addressing work related stress have been included in the study. One is the JDC model proposed by Kaasek [21,22]. The JDC model holds that psychological strains occur when the demand of the job is high, and the job control (decision latitude) and social interactions (social support) are low. Studies have shown that it is a useful model for explaining work stress and its adverse effect on health [23,24]. The other model, known as the effort-reward imbalance (ERI) model, believes that psychological strains happen when people feel that their effort in work is not adequately appreciated. In addition, a personal characteristic i.e. excessive emotional and mental involvement with the work (overcommitment) may aggravate the strain [25-28]. The two models complement each other in that the JDC model deals with the task characteristics and social aspect of the job, while the ERI model touches on the perceived effort-reward imbalance and personal cognitive style of coping with the work. Therefore, factors of both models were included together with demographic factors, factors of lifestyle behavior and additional work conditions.

In this study, we attempted to assess the prevalence of anxiety symptoms among Chinese nurses working in public city hospitals. In addition, we aimed to find out whether demographic factors, lifestyle, work conditions, job content and effort-reward imbalance were associated with the anxiety symptoms.

## Methods

### Participants

A cross-sectional survey was performed in Liaoning Province in northeast China, during April-June 2009. Liaoning Province had a population of 43 million. Seven city hospitals in different areas of the province were randomly selected for the study including six ‘grade one’ hospitals (>500 beds) and one ‘grade two’ hospital (101–500 beds). All nurses in these city hospitals composing the study population were a total of 1807 nurses.

### Data collection

A self-administered questionnaire was distributed to the 1807 participants. 1592 of them returned the filled questionnaires, giving a response rate of 88.1%. Among these, 1437 questionnaires were valid (79.5%), and these nurses became our study participants.

### Measures

#### Demographic variables

Demographic factors included age, length of employment, marital status, education, chronic diseases, and life events. Age was divided in to seven layers:≤25, 26–30, 31–35, 36–40, 41–45, 46–50, ≥51 years. Length of employment was stratified into four layers: ≤4, 5–14, 15–20, ≥21 years. Marital status was categorized as married/cohabitation or single/widow/divorced/separated. Education was divided into three categories: technical nursing school or lower (3 years nursing school after graduation from junior high school), junior college (3 years nursing school after graduation from high school), and bachelor degree or above (5 years college education or higher after high school). Chronic disease was considered as “presence” if any long term illness such as cardiovascular disease, diabetes, arthritis, etc. had ever been diagnosed. Life events referred to the occurrence of death, major disease or unemployment of family members, or offspring’s admission to college or marriage in the last year.

#### Lifestyle

The following variables were included: smoking, alcohol consumption, regular meals, and physical exercise (no exercise, once a week, twice or more a week).

#### Work conditions

Seven factors were considered: (1) hospital grade, (2) job rank, (3) night shift, (4) monthly salary, (5) nurse–patient relationships, (6) job satisfaction, and (7) intention of leaving. Hospital grade was categorized as grade one or grade two according to the Management Criterion of the Ministry of Health in China. Job rank was categorized as head nurse and basic nurse by asking a question: “Are you a head nurse or basic nurse?” Night shift was measured by asking a question: “Do you work night shift (yes/no)?” Monthly salary was divided into 2 categories: <2000, and ≥2001 yuan RMB. Nurse–patient relationships were measured with a single question: “How often does the relationship between the patients and nurses in your work bother you?” with 5 possible answers (never, rarely, sometimes, frequently and always). The response was further divided into three categories: mild (never/rarely), moderate (sometimes) and serious (frequently/always) [29]. Job satisfaction was measured with a simple question: “In general, are you satisfied with your job?” with three possible answers (satisfied, neutral and dissatisfied). Finally, intention of leaving was measured with a question: “Have you ever thought about leaving the current job?” with 3 possible responses (sometimes, often and never). The response was further dichotomized into “yes (sometimes/often) or no (never)”.

#### Job content

A Chinese version of Job Content Questionnaire (JCQ) was used to assess the work stressors and related work environment. It had been reported as having good reliability and validity in Chinese health populations [30]. The questionnaire contains 22 items of three scales. The decision latitude contained two subscales: skill discretion and decision authority, and had 9 items; Scale of psychological job demands contains 5 items; Social support scale includes 2 subscales, supervisor support and coworker support, and contains 8 items. Responses to all the items were scored as 1 meaning complete disagreement and 4 meaning complete agreement. All the scores of the responses for the scales and subscales were dichotomized as “high” or “low” from the median.

#### Effort-reward imbalance at work

This was evaluated with a Chinese version of the effort-reward imbalance (C-ERI) questionnaire. C-ERI was considered a reliable and valid instrument for measuring psychosocial stress at work in Chinese populations, in particular, in Chinese health care workers [31,32]. Basically, the C-ERI questionnaire contains 23 items that measure extrinsic effort (6 items), reward (11 items), and overcommitment (6 items). “Extrinsic effort” refers to demanding aspects of the work environment (time, responsibility, and physical load). “Reward” mentions three aspects of the job: esteem, job promotion and job security. “Overcommitment” measures a person’s motivational pattern of coping with demands characterized by a tendency to be absorbed in work excessively. Responses to “extrinsic effort” and “reward” are scored from 1 to “not stressful” and 5 to “considerably stressful”. “Effort/reward ratio”, which is drawn from the combination of extrinsic effort and reward, was used to indicate “stressful” if this ratio was over 1. Response to “overcommitment” was scored from 1 meaning “complete disagreement” to 4 meaning “complete agreement”. The response was dichotomized as “high” or “low” from the median.

#### Measurement of anxiety symptoms

The Zung Self-Rating Anxiety Scale (SAS) was used to measure anxiety related symptoms of the nurses. SAS was designed to record the presence and quantify the severity of anxiety as a clinical disorder and was evaluated in the original publication [33]. Studies showed that its internal consistency in non-psychiatric and psychiatric samples was adequate with good item-total correlations and good test–retest reliability [34-36]. The Chinese version of the questionnaire has been widely used among Chinese populations and has been found reliable in epidemiological surveys (the Cronbach’s alpha was 0.85) [37,38]. The SAS questionnaire consists of 20 items; each item is scored according to the frequency and duration of the symptoms: none or a little of the time (scored as 1), some of the time (2), good part of the time (3) and most of the time (4). The minimum total raw score is 20, and maximum 80; a high score is indicative of high level of anxiety. Although a cutoff point raw score of 36 was set by the original author to define the anxiety symptoms that were clinically significant [39], studies in the Chinese populations showed the upper limit for the normative populations was a raw score 40 or index score 50 [37]. In this study, we defined the “anxiety symptoms” as when the total raw score became ≥40 [37,38].

### Ethics

Participation of the survey was voluntary. Consent was obtained from each participant. The Committee on Human Experimentation at China Medical University approved the study.

### Data analysis

Among all independent variables, items to which over 95% of individuals have the same responses were not included in the data analysis. Smoking was thereby excluded.

Distribution of anxiety symptoms in categorical variables were tested by the chi-square test. Kappa test was conducted to examine the agreement among categorical variables. Variables with Kappa value ≥ 0.50 were considered as agreement and would be adjusted in multivariate analysis. In this study, no variables were found to be in agreement. Stepwise multivariate logistic regression analysis was used to identify the factors associated with anxiety symptoms. Variables related to anxiety symptoms in univariate analysis (P < 0.25) were entered into the model with adjustment for age. All the statistical analyses were performed with SPSS for Windows (version 13.0), with a two-tailed probability value of <0.05 considered to be statistically significant.

## Results

### Characteristics of the participants

Characteristics of the participants were shown in Tables 1, 2 and 3. All the nurses were female, and the average age of the study population was 35.01 ± 9.33 years (mean ± SD).

**Table 1:** Demographic factors of participants and univariate analysis of factors related to anxiety symptoms (N = 1437)

**Variable**	**Number of participants in different categories No. (%)**	**Number of participants with anxiety symptoms No. (%)**	***P *value**
Demographic factors			
Age (years)			0.714
≤25	310(21.6)	127(41.03)	
26–30	250(17.4)	107(42.8)	
31–35	152(10.6)	65(42.8)	
35–40	333(23.2)	150(45.0)	
41–45	139(9.7)	62(44.6)	
46–50	165(11.5)	68(41.2)	
≥51	88(6.1)	45(51.1)	
Length of employment (years)			0.647
≤4	370(25.7)	159(43.0)	
5–14	310(21.6)	126(40.6)	
15–20	373(26.0)	169(45.3)	
≥21	384(26.7)	170(44.3)	
Marital status			0.658
Single/ Widow/divorced/separated	477(33.4)	204(42.8)	
Married/cohabitation	950(66.6)	418(44.0)	
Education			0.012
Technical nursing school or lower	474(33.0)	180(38.0)	
Junior college	705(49.1)	329(46.7)	
Bachelor degree or above	258(18.0)	115(44.6)	
Chronic diseases			<0.001
Yes	224(15.8)	124(55.4)	
No	1192(84.2)	486(40.8)	
Life events			0.001
Yes	588(41.8)	284(48.3)	
No	820(58.2)	323(39.4)	

**Table 2:** Lifestyle and work conditions of participants and univariate analysis of the factors related to anxiety symptoms (N = 1437)

**Variable**	**Number of participants in different categories No. (%)**	**Number of participants with anxiety symptoms No. (%)**	***P *value**
Lifestyle			
Alcohol consumption			0.083
Yes	84(8.9)	42(50.0)	
No	857(91.9)	345(40.3)	
Regular meals			<0.001
Yes	761(61.7)	290(38.1)	
No	472(38.3)	244(51.7)	
Physical exercise			<0.001
No	798(58.5)	373(46.7)	
Once a week	400(29.3)	154(38.5)	
Twice or more a week	166(12.2)	52(31.3)	
Working conditions			
Hospital grade			0.045
Grade one	1281(89.1)	568(44.3)	
Grade two	156(10.9)	56(35.9)	
Job rank			0.005
Head nurse	160 (11.1)	53(33.1)	
Basic nurse	1277(88.9)	571(44.7)	
Night shift			0.302
Yes	644(44.8)	270(41.9)	
No	793(55.2)	354(44.6)	
Monthly salary (yuan RMB)			0.002
<800	170(11.8)	54(31.8)	
800–1500	594(41.3)	284(47.8)	
1501–2000	532(37.0)	230(43.2)	
>2000	141(9.8)	56(39.7)	
Nurse–patient relationship (irritation)			<0.001
Mild (never/rarely)	844(58.7)	307(36.4)	
Moderate (sometimes)	419(29.2)	200(47.7)	
Serious (frequently/always)	174(12.1)	117(67.2)	
Job satisfaction			<0.001
Satisfied	552(39.1)	169(30.6)	
Usual	578(40.9)	269(46.5)	
Dissatisfied	283(20.0)	174(61.5)	
Intention of leaving			<0.001
Yes (sometimes/often)	766(54.2)	389(50.8)	
No (never)	646(45.8)	218(33.7)	

**Table 3:** Job content and effort-reward imbalance of participants and univariate analysis of factors related to anxiety symptoms (N = 1437)

**Variable**	**Number of participants in different categories No. (%)**	**Number of participants with anxiety symptoms No. (%)**	***P *value**
Job content			
Skill discretion			0.100
High	398(27.7)	159 (39.9)	
Low	1039(72.3)	465 (44.8)	
Decision authority			0.002
High	657(45.7)	256 (39.0)	
Low	780(54.3)	368 (47.2)	
Decision latitude			<0.001
High	653(45.4)	246(37.7)	
Low	784(54.6)	378(48.2)	
Psychological job demands			0.076
High	710(49.4)	325(45.8)	
Low	727(50.6)	299(41.1)	
Supervisor support			0.008
High	312(21.7)	115(36.9)	
Low	1125(78.3)	509(45.2)	
Coworker support			0.003
High	318(22.1)	115(36.2)	
Low	1119(77.9)	509(45.5)	
Social support			<0.001
High	393(27.3)	136(34.6)	
Low	1044(72.7)	488(46.7)	
Effort-reward balance at work			
Effort/reward ratio			0.006
≤1	1059(73.7)	437(41.3)	
>1	378(26.3)	187(49.5)	
Overcommitment			<0.001
High	704(49.0)	371(52.7)	
Low	733(51.0)	253(34.5)	

### Univariate analysis of the factors related to anxiety symptoms

The prevalence of anxiety symptoms in these nurses was 43.4%. Results of univariate analysis of the factors related to anxiety symptoms were shown in Tables 1, 2 and 3.

Among the demographic factors, education, chronic diseases and life events were significantly related to anxiety symptoms, whereas age, length of employment and marital status were not (Table 1).

Among the factors of lifestyle and work conditions, regular meals, physical exercise, hospital grade, job rank, monthly salary, nurse-patient relationships, job satisfaction and turnover intentions were found significantly related to anxiety symptoms, whereas alcohol consumption (*P* = 0.083) and night shift were not (Table 2).

Among the three scales of job content, social support (including coworker support and supervisor support) and decision latitude were significantly related to the anxiety symptoms, but psychological job demand was not (p = 0.076). In the decision latitude (job control), however, the skill discretion subscale was not significantly related. Both scales of the ERI model, the effort/reward ratio and overcommitment, were significantly related to the anxiety symptoms (Table 3).

### Multivariate logistic regression analysis of factors associated with anxiety symptoms

Results of the multivariate logistic regression analysis of factors associated with anxiety symptoms were shown in Table 4. The factors that increased the possibility of developing anxiety symptoms were, in the descending order of odds ratio (OR), lower job rank, overcommitment, chronic diseases and worse nurse-patient relationships. On the other hand, the factors that were found to be protective against developing anxiety symptoms were, in descending order of the protection power (ascending order of OR value), higher social support, lower hospital grade, taking regular meals and higher level of job satisfaction (Table 4).

**Table 4:** Multivariate logistic regression analysis for exploring factors associated with anxiety symptoms

**Variables**	**OR**	**95% CI**
Job rank (basic nurse vs. head nurse)	2.501	1.457-4.293
Overcommittment (high vs. low)	2.018	1.500-2.713
Chronic diseases (yes vs. no)	1.541	1.037-2.291
Nurse-patient relationship (serious vs. mild)	1.434	1.134-1.813
Job satisfaction (satisfied vs. dissatisfied)	0.722	0.579-0.900
Regular meals (yes vs. no)	0.719	0.531-0.974
Hospital grade (grade two vs. grade one)	0.629	0.402-0.985
Social support (high vs. low)	0.573	0.410-0.800

## Discussion

### Prevalence of anxiety symptoms in the nurses

Our study showed that the prevalence of anxiety symptoms was 43.4%. According to reports in the Chinese literature that used SAS for the assessment of anxiety symptoms with a raw score of 40 as the cutoff point, the prevalence of anxiety symptoms in nurses working in the city general hospitals ranged from 31.0% to 64.9% (raw score 37.7-43.9) [40-42], so the results of our study were very representative of the Chinese nurses who were working in the city general hospitals. However, in comparison with the Chinese general population or many other populations, the prevalence of anxiety symptoms in the Chinese nurses was markedly higher. Take teachers for an example. Studies that used the same criterion showed the prevalence in primary and middle school teachers was 5.19% (27/518) and 7.84% (20/255) respectively [43,44], though both studies were limited in the number of sites and the sample size. Furthermore, the prevalence was higher than that in the medical students (12.5%) [38], and was even higher than that in cancer patients (32.7%) in a community [45]. Within the medical health care system itself, it was shown that prevalence of anxiety symptoms in nurses was much higher than that in doctors (21.05%) [46]. Comparison of prevalence of anxiety in nurses between different countries at present stage would be difficult if not at all possible. This was due to the fact that different studies have used different scales for assessment of anxiety, or the same scale but different cut-off values. The issue could be further complicated by the fact that samples in some studies were not representative or simply the sample size was not large enough so that generalization of the results could be doubted. Nevertheless, the high prevalence of anxiety symptoms in the Chinese nurses revealed in this study warrants further investigation.

### Associated factors of anxiety symptoms in nurses

It is believed that identification of specific factors predisposing the nurses to the development of anxiety symptoms would be helpful for further intervention. Therefore, in this study, potential factors including those of the demographic features, lifestyle, work conditions, job content and effort-reward imbalances were analyzed for their association with the anxiety symptoms.

Among all the demographic factors, chronic diseases stood out as the strongest associated factor for anxiety symptoms in nurses (OR 1.541). In comparison with nurses who had no chronic illness, the prevalence of anxiety symptoms in nurses with chronic medical conditions was 14.6% higher, reaching 55.4%. This might not be surprising as there were many studies demonstrating the association of chronic illness with mental disorders [47] or vice versa [48,49]. Life event was also related to the anxiety symptoms, and nurses who have experienced life events had a higher prevalence of anxiety symptoms. In addition, nurses with higher educational backgrounds (junior college, bachelor degree or above) appeared to have higher levels of anxiety than the nurses with lower educational backgrounds (technical nursing school or lower). However, the latter two factors did not contribute significantly when other factors were considered in the logistic analysis.

With respect to the lifestyle, both taking meals regularly and doing physical exercises were found related to the level of anxiety symptoms. However, only taking regular meals contributed significantly in the logistic analysis. The prevalence of anxiety symptoms in nurses who were taking regular meals was 13.6% lower than those who were not (OR 0.719). Interestingly, a recent study has shown that high workplace physical activity and low leisure-time physical activity was associated with anxiety and depression, and improved well-being can be achieved through leisure-time physical activity [50]. These findings suggest that promotion of healthy lifestyle may be helpful for reducing anxiety symptoms in nurses.

All factors of working conditions, except for night shift, were related to anxiety symptoms. Specifically, higher hospital grade, lower job rank, lower monthly salary, irritable nurse–patient relationships, lower job satisfaction and intentions of leaving were found related to anxiety symptoms. In the multivariate logistic analysis, job rank (basic nurse vs head nurse) was shown to contribute most to the anxiety symptoms (OR 2.501); In fact, among all four positively associated factors, job rank was the strongest. As shown in the univariate analysis, the prevalence of anxiety symptoms of the basic nurses was 11.6% higher than that of the head nurse. The finding was somewhat surprising as our previous study showed that nurses considered salary as the most influential factor for their job satisfaction [51]. This result might be a reflection of the nursing management system or style. Just like Chinese society generally, nursing management in Chinese hospitals is hierarchical. The head nurse usually has the power to assign work or even decide how much bonus the basic nurses can get. The basic nurses, on the other hand, usually follow orders and tend to restrain themselves from airing different opinions or proposals. This may cause inward conflict and lead to mental problems such as anxiety. Similar results were found in American nurses working in urban university hospitals [52]. Therefore, communication between the head nurses and basic nurses should be improved. We included nurse-patient relationships as a potential work stressor for nurses. Our study showed that bad nurse-patient relationships was indeed associated with anxiety symptoms (OR 1.434), and the prevalence of the anxiety symptoms increased from 36.4% to 67.2% when the reported irritations the nurses experienced increased from never/rarely to frequently/always. The association of bad nurse-patient with anxiety symptoms may not be surprising, as previous studies had already demonstrated, dealing with difficult patients and their relatives might lead to psychological problems in nurses [53]. However, our study revealed that current nurse-patient relationships are very awkward, because 41.3% of the nurses in our study reported that they sometimes or frequently/always felt irritable about the relationship. The prevalence of anxiety symptoms in the frequently/always group was as high as 67.2%. This should arouse the attention from nursing managers and indicates that prompt intervention may be necessary. On the other hand, lower hospital grade (grade II vs grade I) was negatively associated with anxiety symptoms (OR 0.629). Nurse anxieties were known to be associated with organizational features such as ownership status of the hospitals [54]. The fact that the lower hospital grade was associated with less anxiety symptoms was easy to understand. In China, the community health care system or family medicine is still incomplete. Without proper consultation, most patients would chose to go to higher ranking hospitals for their medical care, so the lower ranking hospitals are usually not as busy or crowded as in the higher ranking hospitals. Nurses working in the lower ranking hospitals may experience less pressure due to fewer patients or less severe/emergent cases, and less patient-nurse conflict. In addition, management in lower grade hospitals may not be as strict as that in the higher grade hospitals, and nurses may not feel the pressure as in the higher grade hospitals. Job satisfaction was also found to be negatively associated with anxiety symptoms (OR 0.722). The prevalence of anxiety symptoms in the nurses who were satisfied with their job (30.6%) was much lower than that of the nurses who were dissatisfied (61.5%). Our findings were in line with that of others [46]. All in all, our findings with factors of work conditions indicated that work conditions impacted on the anxiety symptoms of the nurses. While job rank and hospital grade are more or less institutional characters that can not be corrected, patient-nurse relationships and job satisfaction of the nurses are to a large extent influenced or decided by the management style or the capacity of nurses. The strong association of the latter two factors with the anxiety symptoms suggests that improvement in the two factors may help reduce the anxiety symptoms in the nurses.

Recent studies have shown that factors of JDC and ERI models were associated with mental health of nurses [55,56]. In our study, univariate analysis showed that except for skill discretion and psychological job demands, all the other factors were related to the anxiety symptoms of the nurses. Among these, overcommitment and social support contributed significantly according to the multivariate logistic analysis. While overcommitment was positively associated with anxiety symptoms (OR 2.018), social support was negatively associated (OR 0.573). As a personality trait, overcommitment is considered as a dysfunctional coping style characterized by the inability to withdraw from work obligations. It has been known to be associated with reduced mental health [57]. Our study showed that nurses with higher scores on overcommitment also had much higher prevalence of anxiety symptoms (52.7%) than those with lower scores (34.5%), supporting previous findings. This suggests that psychological consultations and assistance might be helpful for the nurses who had such a personality. On the other hand, higher social support at work was shown to be protective against anxiety symptoms in this study. In comparison with nurses who had lower social support, nurses with higher social support had much lower prevalence of anxiety symptoms (34.6% vs 46.7%). This finding has important implications for the nursing managers and hospital administrators. It implies that building a supportive work environment among the co-workers and supervisors may help reduce nurses’ anxiety symptoms.

## Conclusions

In this study, we demonstrated that 43.4% of Chinese nurses working in public city hospitals had anxiety symptoms. Demographic factors, lifestyle factors and factors of job content and effort-reward imbalance were found associated with anxiety symptoms. Among these factors, lower job rank, higher overcommitment, presence of chronic diseases and worse nurse-patient relationships were positively associated with the anxiety symptoms whereas job satisfaction, taking regular meals, lower hospital grade and higher level of social support at work was all negatively associated. Of course, caution should be exerted in interpreting results of this study as, like limitations in any other cross-sectional studies, a causal relationship between the associated factors and the anxiety symptoms could not be ascertained at this stage. Furthermore, nurses of this study were sampled from city hospitals so conclusions of this study could not be generalized to nurses working in other areas (e.g. rural or remote areas) of China as the working conditions there may be different. Nevertheless, comparison of anxiety symptoms in nurses with other populations strongly supports the effective representation of the sample for nurses working in city hospitals. In this sense, the fact that a large proportion of Chinese nurses had anxiety symptoms warrants immediate attention from nursing managers and hospital administrators, with further investigation and intervention needed. In addition, results of this study suggest that proper counseling, promotion of healthy lifestyle practices and improving social environments of the work place may be helpful for reducing or preventing the anxiety symptoms of the nurses.

## Competing interests

The authors declare they have no competing interests.

## Authors’ contributions

YG and LW conceived and designed the study. LW, JW and HW performed the data collection. BP, JW and HW performed the data analysis. YG and BP drafted the manuscript. YG, WS, BP and LW made critical revision to the paper for important intellectual content. WS and LW provided statistical expertise. YG and LW supervised the study. All authors read and approved the final manuscript.

## Pre-publication history

The pre-publication history for this paper can be accessed here:

http://www.biomedcentral.com/1471-244X/12/141/prepub
